# Patient, family and provider experiences with a very low-carbohydrate approach for type 1 diabetes

**DOI:** 10.4102/jmh.v9i1.137

**Published:** 2026-03-25

**Authors:** Svetlana Azova, Erin Gordon, George M. Edwards, Owen Henn, David S. Ludwig, Katharine Garvey, Belinda S. Lennerz

**Affiliations:** 1 Division of Endocrinology, Boston Children’s Hospital, Boston, United States of America; 2 Department of Pediatrics, Harvard Medical School, Boston, United States of America; 3 Division of Gastroenterology, Hepatology and Nutrition, Boston Children’s Hospital, Boston, United States of America; 4 UT Southwestern Medical Center, Dallas, United States of America; 5 Hydrow, Inc., Boston, United States of America; 6 Steno Diabetes Center, Copenhagen, Denmark

**Keywords:** medical nutrition therapy, survey study, qualitative research, type 1 diabetes, very low-carbohydrate diet

## Abstract

**Background:**

Adoption of a very low-carbohydrate (VLC) approach to aid in type 1 diabetes (T1D) management has recently gained momentum; however, there is limited research to guide its optimal implementation and concerns about sustainability and safety.

**Aim:**

This study characterises the lived experiences of adult patients and caregivers of children with T1D who follow a VLC approach and patients’ diabetes medical providers (DMPs).

**Setting:**

Online surveys were fielded internationally to members of the Facebook group TypeOneGrit who follow the VLC approach.

**Methods:**

We performed applied thematic analysis of data based on responses to six open-ended survey questions. Multidisciplinary consensus coding was conducted, culminating in iterative higher-level qualitative analysis to generate a thematic depiction of participant experiences.

**Results:**

A total of 931 responses from 155 adult patients, 112 caregivers and 61 DMPs were included. Analysis yielded four cluster themes: (1) Predominantly negative patient and caregiver experiences within the medical system with regard to the VLC approach, (2) mainly positive patient and caregiver experiences with the VLC approach despite challenges, (3) desire for widespread dissemination of information on the VLC approach and (4) acknowledgement of the positive attributes of the VLC approach and/or patient or family by DMPs, amid DMP concern over potential or encountered adverse outcomes.

**Conclusion:**

This study produced valuable insights on the perceptions and experiences of adult patients, caregivers of children with T1D and patients’ DMPs with regard to the VLC approach.

**Contribution:**

The VLC approach can be a viable adjunctive method for T1D management but requires increased understanding and support.

## Introduction

Despite acknowledgement that individualised medical nutrition therapy is an important component of type 1 diabetes (T1D) care in both children^1,2^ and adults,^3,4^ there continues to be a paucity of robust data on optimal and acceptable nutritional management in T1D. Several studies have documented glycaemic^5,6,7,8,9,10,11,12^ and other benefits (e.g. improved quality of life^6^) of a low-carbohydrate approach in T1D management. Historically, carbohydrate restriction, with variable recommended intake of total calories, protein and fat, was the only available treatment for people with T1D capable of prolonging life expectancy prior to the discovery of insulin.^5^ With the advent of insulin therapy, research into optimal dietary carbohydrate composition declined. Higher carbohydrate eating plans gained favour and made their way into guidelines by major professional diabetes organisations, including the American Diabetes Association (ADA)^3^ and the International Society for Paediatric and Adolescent Diabetes.^2^ Recently, the low-carbohydrate approach has regained popularity among a growing number of patients with T1D, especially following more widespread published reports of glycaemic benefits in this population.^5,13^ There is, however, limited research to guide optimal implementation of this approach, as well as concerns about sustainability and safety, including potential increase in cardiovascular risk factors and compromised growth in children,^2,5,14^ warranting further investigation.

To investigate objective and subjective experiences with a very low-carbohydrate (VLC) approach, a survey study among adult patients and caregivers of children with T1D who self-identified as following this way of eating was conducted.^13^ All participants were part of an international Facebook group who subscribe to the dietary recommendations and diabetes management strategies put forth by Dr. Richard Bernstein in his book *Dr. Bernstein’s Diabetes Solution*.^15^ Participants’ diabetes medical providers (DMPs) were also surveyed to corroborate objective information and lend their own perspectives on their patients’ management approach.^13^ Previously published results of the study documented an average consumption of 36 ± 15 g of carbohydrate per day and exceptional glycaemic outcomes among adults and children with T1D, with a mean haemoglobin A1c (HbA1c) of 5.67% ± 0.66% and a low rate of complications. In addition, adult patients and caregivers of children with T1D assigned a high rating to overall patient health and reported satisfaction with diabetes management.^13^ However, there was a discrepancy in the status of the perceived relationship and support between: (1) adult patients and caregivers of children with T1D, and (2) DMPs. Patients and caregivers expressed a more negative view about the status of the relationship with DMPs and overall low satisfaction with professional diabetes care. Concerningly, 27% of participants reported not discussing their dietary management strategies with providers. Only 49% of those who did discuss their way of eating felt like the providers were supportive. Qualitative comments solicited during the survey may shed light on reasons behind this limited therapeutic alliance and help inform strategies to build trust and improve medical oversight for those who choose to follow a VLC approach for management of their T1D.

Here, we describe and evaluate subjective experiences with a VLC approach among adult patients, caregivers of children with T1D and patients’ DMPs, utilising qualitative data provided by survey respondents during the initial study.^13^ The goal was to enrich our understanding of how individuals, families and DMPs think about a VLC approach for the management of T1D. These insights may help inform future studies aimed at assessing the efficacy, safety, feasibility and sustainability of a VLC approach for the management of T1D.

## Research methods and design

### Study design, setting and selection of participants

An online survey was fielded between September and November of 2016 to collect responses from adult patients and caregivers of children with T1D who are members of the Facebook group TypeOneGrit. This online community was formed by individuals with T1D who follow the low-carbohydrate approach and diabetes management strategies described by Dr. Bernstein in his book *Dr. Bernstein’s Diabetes Solution*.^15^ In addition, a secondary survey of DMPs was conducted to collect confirmatory medical information, as well as their perceptions regarding their patients’ diabetes management approach. Exclusion criteria for patients were following a VLC approach for less than 3 months and/or not requiring insulin at the time of the survey. A detailed description of the overall survey design and participant selection was previously published.^13^

### Data collection

Four open-ended questions were included in the survey for adult patients and caregivers of children with T1D and two in the survey for DMPs (Box 1). In the survey for adult patients and caregivers of children with T1D, participants were asked to describe both their experiences with the VLC approach and DMPs. In addition, participants who indicated that they did not openly discuss their way of eating with providers via a preceding multiple-choice question were asked to describe their concerns. Lastly, a question eliciting suggestions for how the VLC approach can be expanded to the wider audience was included.

In the survey for DMPs, participants were asked to comment on how they felt about their patients’ diabetes management approach. The VLC approach was not explicitly referenced as not all providers may have been aware of their patients’ dietary strategy, and the study team did not want to inadvertently disclose that information. In addition, providers were given the opportunity to include any additional comments that they desired.

### Data analysis

We utilised applied thematic analysis,^16,17,18,19^ informed by the phenomenological lens,^20,21^ to analyse the collected qualitative data. The multidisciplinary research team, composed of two paediatric endocrinologists, a registered dietitian and a predoctoral trainee, read through all free-text responses. The team then used a combination of inductive and deductive approaches to code the data in first-level analysis. Initially, utilising insights from the responses, the research team created a separate codebook for each question based on emergent codes (inductive coding). Team members met and compared the codes, engaging in thoughtful discussions aimed at reconciling the codebooks. Following reconciliation, two co-authors (SA and EG), who received appropriate training, independently (i.e. blinded to the other coder) coded all free-text responses, one question at a time, while taking detailed notes regarding their observations. Once both team members completed coding each question, they met and reviewed the applied codes, resolving any differences, as able. In addition, the team members amended their codebooks based on their notes and observations and then recoded the data based on the updated codes (deductive coding). Afterwards, they reviewed and compared their codes once more to reconcile any differences. This required comprehensive data triangulation to achieve thematic data saturation. Once this process was completed for each of the six questions, any discrepancies and disagreements in the coding were broken by the senior author. The two co-authors (SA and EG) then met to finalise the reconciliation of the codes for all six questions.

Following the initial coding, the two co-authors (SA and EG) utilised Dedoose software (Version 9.0.107, SocioCultural Research Consultants, LLC, Los Angeles, CA)^22^ for the next steps of thematic grouping and analysis. The coding team met multiple times to condense the codes into categories in a second-level coding analysis. Each question was initially analysed separately. However, as overlapping topics started to arise between: (1) Q1 and Q3, and (2) Q5 and Q6, findings from those questions were combined and condensed accordingly. Finally, in third-level analysis, categories were condensed further to identify final, key themes that best described and summarised the data. Analysis of data was conducted in aggregate and also by participant type (adult patients versus caregivers of children with T1D) to determine whether there were any differential findings. The research team kept a detailed audit trail of the survey response review and analysis process that led to the emergence of the final themes.

### Ethical considerations

Ethical clearance to conduct this study was obtained from the Boston Children’s Hospital Institutional Review Board on 23 August 2016 (No. IRB-P00022864). All procedures performed in studies involving human participants were in accordance with the ethical standards of the institutional and/or national research committee and with the 1964 Helsinki Declaration and its later amendments or comparable ethical standards. Written informed consent was obtained from all individual participants involved in the study. Only deidentified data were included in the analysis and publication, maintaining participant confidentiality.

## Results

### Participant characteristics

A total of 931 responses across the six open-ended questions from 155 adult patients and 112 caregivers of children with T1D, as well as 61 DMPs, were included in the initial coding process. Table 1 shows the total number of coded responses included in the final analysis (775), categorised by participant type for each question. A detailed description of the participant characteristics has been previously published.^13^

### Thematic analysis

**FIGURE 1:**
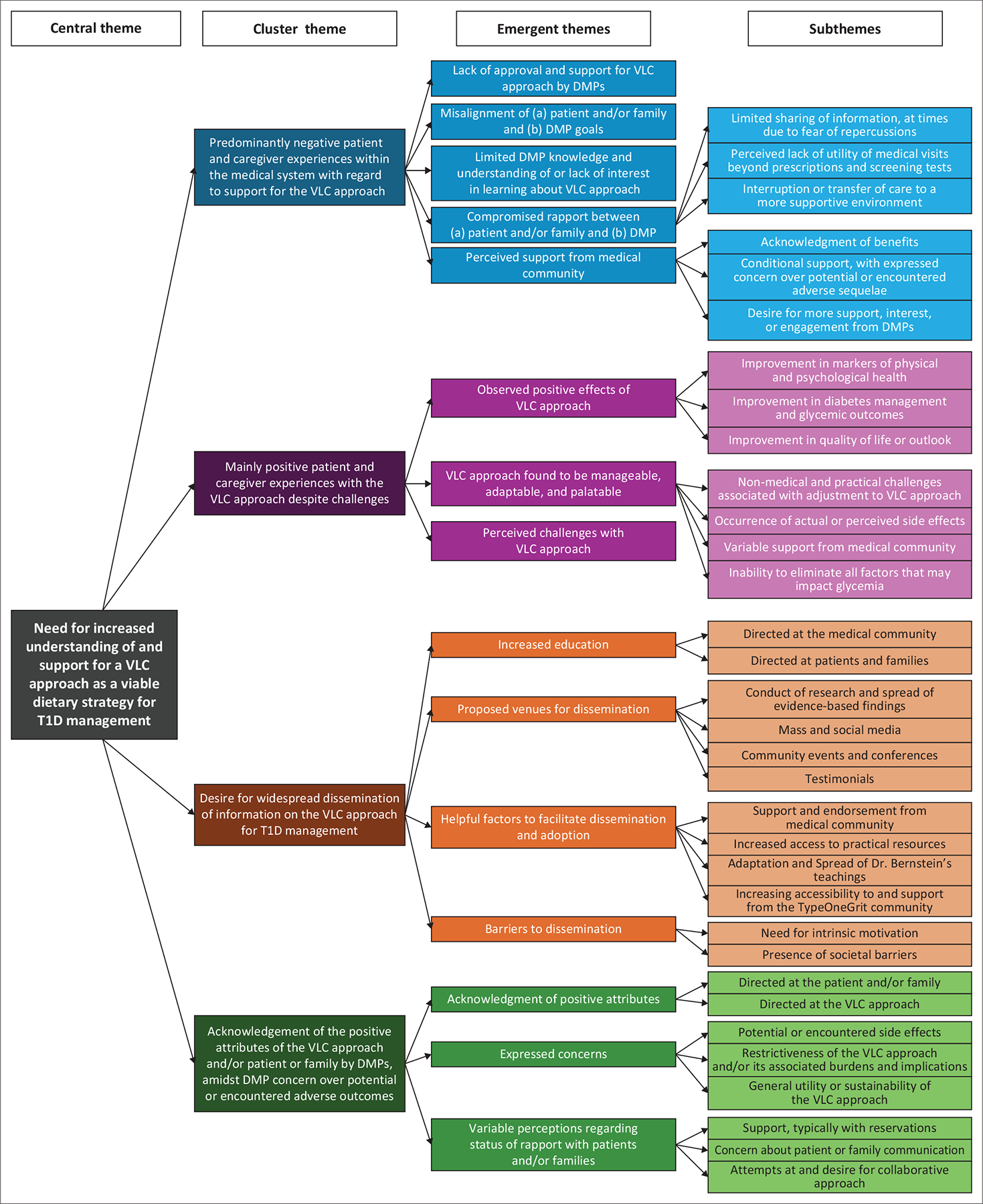
Thematic depiction of patient, caregiver and diabetes medical provider experiences with a very low-carbohydrate approach for type 1 diabetes management. DMP; diabetes medical provider; T1D, type 1 diabetes; VLC, very low carbohydrate.

Our iterative thematic analysis generated four key cluster themes across the open-ended survey questions, depicted in Figure 1: (1) Predominantly negative patient and caregiver experiences within the medical system with regard to support for the VLC approach, (2) mainly positive patient and caregiver experiences with the VLC approach despite challenges, (3) desire for widespread dissemination of information on the VLC approach for T1D management, and (4) acknowledgement of the positive attributes of the VLC approach and/or patient or family by DMPs, amid DMP concern over potential or encountered adverse outcomes. The central theme uniting all of these was the overall need for a better understanding of the efficacy and safety of the VLC approach for the care of children and adults with T1D and increased support for its integration into T1D management.

#### Theme 1: Predominantly negative patient and caregiver experiences within the medical system with regard to support for the VLC approach

When asked to describe their experiences with DMPs, many respondents cited actual or presumed lack of support for the VLC approach. Some endorsed more positive interactions, however, often in comparison to negative experiences with other providers. Analysis of data revealed largely similar perceptions among adult patients and caregivers of children with T1D, except where indicated.

Patients and caregivers described largely negative interactions with DMPs when it came to the VLC approach. Many providers were perceived as not agreeing with, approving of or supporting this dietary strategy, especially for children with T1D:

‘The endocrinologists, NPs [*nurse practitioners*], CDEs [*certified diabetes educators*], and dieticians [*sic*] are very antagonistic toward the diet and, initially, toward us. Now I feel as though that [*sic*] tolerate us and our decisions, but they are not for it at all and continually tell us they expect it to fail.’

(ID479, child patient, 6 years old, female [*Caregiver response*])

In addition, patient and caregiver goals often did not align with those of DMPs, who sometimes expressed concern over potential or encountered side effects:

‘Going to the doctor stresses me out. It’s always an argument about my healthcare. They push statins, ace [*angiotensin-converting enzyme*] inhibitors, insulin pumps and hormonal birth control on me at every visit. I refuse and tell them I want to do it through diet and exercise.’

(ID168, adult patient, 27 years old, female)

Others expressed frustration with limited DMP knowledge and understanding of the VLC approach and/or lack of interest in learning more about it, at times leading to a perceived lack of utility of medical visits beyond prescriptions and screening tests:

‘I really just want my lab results & prescriptions updated by my provider. I don’t find it useful to talk about my lifestyle, but if he asked I would gladly answer.’

(ID10, adult patient, 37 years old, female)

‘They are not very knowledgeable. They have not helped us figure out one single problem that we have encountered in our child’s diabetes management. We do not seek their advice. We dutifully go to them 3–4 times a year to get an a1c [*HbA1c*], to be in compliance for insurance purposes, and to get our prescriptions renewed.’

(ID97, child patient, 10 years old, male [*Caregiver response*])

These negative interactions often undermined the relationship between: (1) adult patients and caregivers of children with T1D, and (2) the medical team, which, in addition to a lack of perceived utility, led to:

- Limited sharing of information, at times because of fear of repercussions:

‘We do not offer information because we understand that how we eat, how we manage, fall outside their standard operating procedures. We do not wish to risk investigations (CPS [*Child Protective Services*]) into how we care for our child simply because our diet is different.’

(ID97, child patient, 10 years old, male [*Caregiver response*])

- Interruption or transfer of care to a more supportive environment:

‘We fired endo back in 2013 because she believed low carb would harm my child. .she [*sic*] also believed an a1c [*HbA1c*] in the 5s was unrealistic. We now found a dr [*doctor*] that teaches low carb and the Bernstein approach to all of her patients. She is amazing and has even visited and learned from dr. [*sic*] Bernstein.’

(ID216, child patient, 8 years old, male [*Caregiver response*])

#### Theme 2: Mainly positive patient and caregiver experiences with the VLC approach despite challenges

When asked to describe their experiences with the VLC approach in general, many participants (both adult patients and caregivers of children with T1D) endorsed positive outcomes in health and factors related to diabetes management, culminating in improvement in quality of life. The VLC approach was considered manageable by participants, although some identified challenges.

Participants identified considerable actual or perceived positive effects associated with the VLC approach in the following areas:

- Improvement in markers of physical and psychological health, including energy, sleep, weight, hunger, athletic endurance, mental acuity and mood symptoms:

‘Since following the LCHF [*low-carbohydrate, high-fat*] diet … I no longer have an afternoon slump/nap, I no longer feel the need to eat every few hours, I can skip meals without distress and/or hunger, great lean muscle mass, my body weight is stable, I no longer have cravings and I sleep better.’

(ID61, adult patient, 46 years old, male)

‘More energy, less mood swings. Less stomach aches and headaches.’

(ID133, child patient, 8 years old, female [*Caregiver response*])

- Improvement in diabetes management, including glycaemic outcomes, burden of care and fear about acute and chronic complications:

‘I’ve never had better control over my blood sugars. I wish I would have started this diet years ago. I now feel like I’m going to live a normal healthy life instead of a shortened life riddled with complications from high bloods sugars.’

(ID424, adult patient, 37 years old, male)

‘Only way we have managed to keep bgl [blood glucose levels] in normal range. Has taken the fear away of extreme highs and lows. Don’t worries [*sic*] about child dying overnight from hypoglycemia … Much easier to care for our child …’

(ID196, child patient, 6 years old, male [*Caregiver response*])

- Overall improvement in quality of life and outlook as a result of optimised health and diabetes-related outcomes:

‘Changed our lives for the better by being a way to stabilise his levels, more quality sleep, less stress, less hypos, improved health – physically and mentally, freedom with food, generally a more positive approach to sustain wellbeing over a lifetime. Gave us hope of having as close to a normal life again for our son.’

(ID467, child patient, 12 years old, male [*Caregiver response*])

Many participants found the VLC approach to be manageable, adaptable, not overly restrictive and acceptable or palatable. This perception was more commonly expressed by caregivers of children with T1D, many of whom endorsed adoption of the VLC approach by the whole family:

‘My child eats a healthy diet of non starch vegetables with delicious protein, nuts, and cheese. We do full fat. Our entire family eats healthier and has cut out starches and grains from family meals. Do not feel sorry for him or his diet is restricted[.] [*We*] have delicious and yummy treats. We make low carb treats such as ice cream, smores, marshmallows, graham cracker, bread[,] etc [*sic*].’

(ID466, child patient, 3 years old, male [*Caregiver response*])

Still, a number of participants identified several challenges and shortcomings associated with the VLC approach, in addition to the variable support from the community. These can be divided into the following categories:

- Non-medical practical challenges related to the restrictiveness of the approach, resultant anxiety over blood glucose fluctuations with deviations from the eating plan, burden of management in different settings and the need for certain adjustments in lifestyle:

‘So good to be off the rollercoaster. I don’t follow LCHF [*low-carbohydrate, high-fat*] 100%, I make exceptions around holidays[,] etc. The inevitable resulting highs/lows make me anxious and I don’t want to go to bed until I’m convinced my blood sugar is stable, which can take hours.’

(ID282, adult patient, 33 years old, female)

‘Works well mostly. A bit hard to eat different food in school, and even in some private situations. At home, no problem at all.’

(ID326, child patient, 11 years old, female [*Caregiver response*])

- Occurrence of side effects attributed to the VLC approach, predominantly among adults with T1D:

‘I have tried to go under 50 grams of carb a day as per Dr Bernstein’s method, however[,] it is not enough for me. I felt very tired and unwell. Ketosis did not agree with me. I have since increased carb intake to about 50 per day and have felt much better.’

(ID471, adult patient, 22 years old, female)

- Inability of the VLC approach to eliminate other factors that may impact glycemia:

‘My daughter’s numbers through out [sic] the day is [are] in range. Her last a1c [*HbA1c*] was higher due to an illness and overnight highs due to hormones. If we could control those[,] she would be a perfect 70.’

(ID374, child patient, 5 years old, female [*Caregiver response*])

#### Theme 3: Desire for widespread dissemination of information on the VLC approach for T1D management

When asked about how the VLC approach might be expanded to a larger audience, both adult patients and caregivers of children with T1D emphasised the need for increased efforts in educating the public on this dietary strategy for T1D management and provided specific suggestions for how to facilitate dissemination of the relevant information. Targets for the proposed education include:

- The medical community:

‘I think it starts in the medical school. Nutrition is a big part of this disease and the med students should be made aware of the consequences of high carbs and the dangers they impose on Diabetics … The ADA [*American Diabetes Association*] need [*sic*] to take note of the advances that we are having with low carb and make Doctors aware that there is a better way through medical conferences or training sessions.’

(ID108, adult patient, 60 years old, female)

- Patients and families, especially at the time of T1D diagnosis:

‘I would love on diagnosis for new T1’s to be at least given a choice and be educated about the low carb … approach and be told about the positive results this approach gives and then have a medical professional support this transition who understands it … [*We*] need to have access to support from medical professionals so patients can get support and help when they need it rather than having to shoulder all the decisions themselves.’

(ID239, child patient, 14 years old, female [*Caregiver response*])

Participants identified several venues for education and dissemination of information, including:

- Conduct of research and spread of evidence-based findings:

‘It is crucial that formal studies/research are published to support the effectiveness of [*VLC approach*] so that this way of eating could at least be mentioned at diagnosis …’

(ID171, child patient, 8 years old, male [*Caregiver response*])

- Mass and social media:

‘A massive ad campaign; several approaches come to mind: benefit of stable bg [blood glucose] in learning/growing vs carb & cover; side effects of high insulin over time …’

(ID167, adult patient, 54 years old, female)

- Community events and conferences:

‘I would love to see workshops in every major city. I think people with type 1 are often looking for support and to connect with others and we should tap into that. By offering organised meetings or groups, showing people the information, the graphs and studies while they’re physically present and can ask questions and participate. That way it’s also offering support …’

(ID111, adult patient, 33 years old, female)

- First-person testimonials:

‘It’s expanded this much by personal experience of families connecting with others and helping each other … Grit [*TypeOneGrit*] is more than a way, it’s community. We need GRIT [*TypeOneGrit*] camps for families and children’s sleep away camps.’

(ID24, child, patient, 7 years old, male [*Caregiver response*])

In addition to the proposed methods above, participants identified additional factors that might be helpful in facilitating the successful adoption of the VLC approach on a broader scale:

- Getting support and endorsement from the medical community, including DMPs and professional organisations, the latter more commonly advocated for by the adult participants:

‘By getting more mainstream CDEs [*certified diabetes educators*] to champion thisway [sic] of eating & getting it into medical recommendations for diabetes management.’

(ID10, adult patient, 37 years old, female)

- Increasing access to practical resources:

‘… Also supplying newly diagnosed with some recipes to get them started and having someone cook some basic recipes with them would also help them make the transition easier …’

(ID239, child patient, 14 years old, female [*Caregiver response*])

- Adaptation and dissemination of Dr. Bernstein’s teachings, including increasing accessibility to and support from the TypeOneGrit community:

‘… Dr Bernsteins [*sic*] book ‘Diabetes Solution’ should be given to every newly diagnosed T1D family to read and discuss with their diabetes management team …’

(ID19, adult patient, 49 years old, female)

‘TYPEONEGRIT [*TypeOneGrit*] online needs to take a more approachable stance. It’s known to be militant and exclusive. This information should be available to all, but I know plenty of people who feel attacked by the GRIT [*TypeOneGrit*] group. Dr. Bernstein has an incredible program, and i [*sic*] know GRIT [*TypeOneGrit*] means well. Lets [*sic*] just focus on helping rather than putting people down for not knowing better. We’ve all been in the learning position, where they are now.’

(ID30, adult patient, 39 years old, female)

Lastly, a small but important minority of adult patients and caregivers of children with T1D acknowledged that education and dissemination alone may not be enough, citing the need for motivation and effort on behalf of the patient and family and the presence of larger societal barriers to implementation of the VLC approach:

‘… People who aren’t looking for a better solution than the “standard of care” are not very likely to adopt this WOE [*way of eating*] because it does take more effort. If more people in the medical establishment would get on board that this is a safe and effective alternative, then it could be presented as an option to those who would take advantage of it.’

(ID388, child patient, 8 years old, male [*Caregiver response*])

#### Theme 4: Acknowledgement of the positive attributes of the VLC approach and/or patient or family by DMPs, amid DMP concern over potential or encountered adverse outcomes

Diabetes medical providers (DMPs) were asked to share feelings about their patients’ diabetes management approach and to provide additional comments. In their responses, DMPs highlighted benefits of the patients’ diabetes care strategies and/or shared positive perceptions about the actual adult patient or family of a child with T1D. However, many also expressed concern over potential or encountered side effects and complications. Those who specifically acknowledged or alluded to the VLC approach in their responses were more likely to express concerns over side effects. Diabetes medical providers (DMPs) had variable perceptions regarding the status of their relationship with patients and families, but many expressed the desire for a collaborative approach to management.

Diabetes medical providers (DMPs) shared positive perceptions, regarding: (1) their adult patients or families of children with T1D, and/or (2) the VLC approach and its perceived effects. These positive perceptions were more common on behalf of adult participants and when all responses were included in the analysis:

- Positive perceptions regarding patient or family:

‘He is an excellent example of what can be done in a well motivated patient. He tries to keep his Carbohydrate intake to < 10g/meal. He is also a marathon runner.’

(ID109, adult patient, 56 years old, male [*Response on behalf of patient*])

- Positive perceptions regarding the VLC approach and its perceived effects:

‘Very well controlled with last a1c [*HbA1c*] 6.3 and 5.6%[,] Respectively. No known complications of DM [*diabetes mellitus*] … She is currently eating a Very low carb diet ….’

(ID312, adult patient, 52 years old, female [*Response on behalf of patient*])

At the same time, DMPs, particularly those who explicitly acknowledged or alluded to the VLC approach in their responses, expressed concerns about:

- Potential or encountered side effects, especially on behalf of children with T1D:

‘His diabetes control in terms of HbA1c is excellent[,] and I applaud his parents [*sic*] efforts and commitment to their son. However[,] I am concerned about the effects of extreme carbohydrate restriction on growth, cognitive function and psychological wellbeing long-term. His height has decelerated (growth velocity is 4.3 cm/year)[,] and his diet is very high in sodium, fat, protein and several vitamins. I am concerned about the long-term cognitive and physical effects of extreme carbohydrate restriction and consequent excess of the other macronutrients[,] and I have recommended that this be liberalised with insulin cover.’

(ID171, child patient, 8 years old, male [*Response on behalf of patient*])

- Restrictiveness of the approach and/or its associated burdens and potential implications, predominantly on behalf of the child patients:

‘I am concerned that the family is overly restricting the carbs. I am concerned that this is going to lead to an eating disorder in the future so significant rebellion when the patient becomes a teenager.’

(ID76, child patient, 9 years old, female [*Response on behalf of patient*])

- General utility or sustainability of the approach:

‘I’m uncertain how sustainable this child’s nutritional approach is over time. I’m concerned about the lipid profile[,] but there are no good evidence-based recommendations for this age group … I am still uncertain as to the long-term viability of her approach or the child’s long-term willingness to adhere …’

(ID183, child patient, 3 years old, female [*Response on behalf of patient*])

Finally, DMPs had variable perceptions about the status of their relationship with patients and families, including:

- Support of patient or family and/or openness to the VLC approach:

‘I am very supportive of the approach to management. [*sic*] as it seems sensible[.] The only reservation I have is this type of low carb, high protein diet is not studied adequately in children to know of [*sic*] any longer term poor growth/nutritional outcomes will, [*sic*] occur, however[,] the family understand [*sic*] this as well.’

(ID54, child patient, 7 years old, female [*Response on behalf of patient*])

- Concern about patient or family communication and rapport with the medical community, with a desire for a more collaborative approach to diabetes care:

‘Although the child is growing well, I’m not sure he is getting enough CHO [*carbohydrate*] for his energy requirements. The family does not share details about the nutrition choices and amounts, or whether they use any supplements, or whether the child may be ketotic (or how often). The family does not interact well with the non-MD [*medical doctor*] diabetes educators and staff. The family is very diligent and careful, and I feel the child is safe from a glucose control perspective. I wish they could have a more collaborative approach with us. More openness and transparency is [*sic*] safer, and we all learn.’

(ID13, child patient, 7 years old, male [*Response on behalf of patient*])

## Discussion

This qualitative analysis of free-text survey responses elucidated comprehensive, wide-ranging experiences regarding the use of a VLC approach for diabetes management in both adults and children with T1D. We identified four cluster themes that deserve further attention, exploration and intervention: (1) Predominantly negative patient and caregiver experiences within the medical system with regard to the VLC approach, (2) mainly positive patient and caregiver experiences with the VLC approach despite challenges, (3) desire for widespread dissemination of information on the VLC approach, and (4) acknowledgement of the positive attributes of the VLC approach and/or patient or family by DMPs, amid DMP concern over potential or encountered adverse outcomes. While certain lived experiences and perceptions may have differed among individual participants, there was a central theme of the need for increased understanding of and support for a VLC approach as an adjunctive dietary strategy to support T1D management.

The low-carbohydrate and VLC eating plans have recently been acknowledged as possible options in type 2 diabetes (T2D), albeit with mixed findings regarding glycaemic and cardiometabolic outcomes.^3,4^ However, their potential role in T1D management,^3^ and especially in children,^2,4^ remains undefined despite literature suggesting its potential benefits in individuals with T1D, including with regard to glycemia^5,13^ and metabolic parameters.^23^ Furthermore, as dysglycaemia in T1D has been linked to structural and functional brain abnormalities,^24,25,26,27^ the VLC approach may be associated with potential long-term neurocognitive benefits because of its positive effect on glycemia. However, concerns about the safety and sustainability of the VLC approach remain. The VLC approach is also currently not recommended for diabetes management in the setting of pregnancy and lactation, kidney disease and presence of or concern for disordered eating.^4^ One of the main reservations about using the VLC approach for T1D management, especially in children, is concern over side effects, including potential for increased risk of ketoacidosis, hypoglycaemia, growth impairment, significant dyslipidaemia, disordered eating and psychological ramifications associated with severe carbohydrate restriction.^2,5,14^

Concern over side effects was acknowledged in this study, especially by DMPs who explicitly referenced the use of the VLC approach by their patients. These concerns, particularly in the paediatric population, raise important medical and ethical implications surrounding the principles of informed consent, developmental needs and both short- and long-term health and safety of the child. However, although a minority of adult patients (9%) and caregivers of children with T1D (1%) reported adverse consequences attributed to the VLC approach (e.g. abnormal lipid levels, side effects associated with ketosis), the general experience of these individuals remained overwhelmingly positive. Many participants reported improvements in physical (23%), psychological (17%) and overall health (21%), including energy levels, sleep quality, weight and hunger cravings (lending support to the carbohydrate-insulin model^28^), athletic endurance, mental acuity and mood symptoms. Several (7%) dispelled concerns about adverse outcomes, including significant dyslipidaemia in adult patients and growth impairment and sense of deprivation or restrictiveness in children. This is consistent with adult patients and caregivers of children with T1D assigning a high rating to overall patient health on quantitative analysis of the data.^13^

In addition, these participants also reported improvement in outlook and quality of life, often translating to the whole family, in this study.

As the above findings were based on subjective accounts by a self-selected group of individuals, they should be interpreted with caution, especially amid DMP reports of adverse outcomes. However, several prior studies in T1D also failed to show adverse sequelae associated with the low-carbohydrate approach, such as diabetic ketoacidosis (DKA), severe hypoglycaemia, significant dyslipidaemia/risk of cardiovascular disease and renal impairment.^5,6,7,8,9,12,13,29^ That said, because these studies included varying levels of daily carbohydrate intake restriction, their applicability to VLC eating plans with more stringent restrictions is unclear. In addition, when it comes to growth impairment, while there have been some reports in paediatric T1D that noted a decrease in growth velocity with carbohydrate restriction,^14^ much of the prior concern came from studies in youth with epilepsy on ketogenic diets. In that population, additional factors, including more extreme macronutrient composition, other medical comorbidities and medication effects, may be confounding the association between carbohydrate restriction and growth impairment.^30,31^ Furthermore, even children with epilepsy on ketogenic diets may be managed successfully, without significant effects on their growth, with nutritional optimisation.^32^ Further rigorous, long-term studies are needed to assess the safety of the VLC approach for T1D management and how it could be optimised with adequate support and guidance (i.e. via a focus on nutrient density) to mitigate the risk of adverse sequelae, including potential growth impairment in children.

Despite mixed reports and perceptions when it comes to potential side effects, there was an almost universal acknowledgement of positive diabetes-related outcomes among people with T1D following a VLC approach. Adult patients and caregivers of children with T1D reported significant improvements in glycemia, a decrease in the burden associated with diabetes care and a reduction in the fear of both acute and chronic complications. DMPs, likewise, often recognised glycaemic benefits of the VLC dietary strategy, albeit sometimes amid concern for adverse events, such as greater occurrence of hypoglycaemia. Satisfaction with glycaemic outcomes was also acknowledged by all three participant types in the original study.^13^ Quantitative findings previously published from this survey revealed a mean HbA1c of 5.67% ± 0.66% and a low rate of complications, including hospitalisations for DKA and hypoglycaemia.^13^

Other observational studies and case reports and series likewise documented exceptional glycaemic outcomes among patients with T1D following the low-carbohydrate approach^5,7,8,9,10,11^; however, these findings should be interpreted with caution because of the non-rigorous study designs and small sample sizes. Two recent clinical trials assessing the efficacy of the low-carbohydrate approach in individuals with T1D were conducted. In a non-randomised single-arm trial, the low-carbohydrate eating plan was associated with significant improvements in HbA1c and glycaemic time-in-range (TIR) and reduction in daily insulin use in adults with T1D.^6^ A parallel randomised controlled trial (RCT) comparing a low-carbohydrate vs Mediterranean eating plan in adolescents and young adults with T1D found more significant improvements in HbA1c and glycaemic TIR with the former, without increased risk of hypoglycaemia.^12^

In addition to glycaemic benefits, many adult patients and caregivers of children with T1D found the VLC approach to be manageable, acceptable, palatable and adaptable to the whole family. This view was more commonly expressed by the caregivers, seemingly to dispel concerns that this dietary strategy is overly restrictive for children with T1D, a worry shared by some DMPs (41%). It is important to note that perceptions regarding the manageability, acceptability and palatability of the VLC approach on behalf of the paediatric patients came from their caregivers and thus may not accurately reflect the viewpoints of the children themselves. It would thus be helpful to elicit direct experiences from the children themselves, especially as much concern regarding VLC management is directed to this population. It is also notable that the majority of the adult patients and caregivers of children with T1D in this study identified themselves as being in the upper- and middle-income class (92%), and this may have played a role in their overall predilection towards describing the VLC approach as being manageable and adaptable. In addition, the majority of the adult patients and caregivers of children with T1D were white, non-Hispanic (88%), and all were English speaking. A recent qualitative analysis of Hispanic caregivers’ experience with T1D described culturally-based nutritional challenges, including baseline difficulties with acculturation and integration into the American society and way of eating.^33^ Thus, sociodemographic factors and availability of and access to the necessary supports and resources should be taken into consideration when making decisions about the incorporation of a VLC approach into the daily routine.

Despite an overall positive perception regarding the adaptability and acceptability of the VLC approach, some patients and caregivers in this study also identified practical challenges associated with this dietary strategy. These included occasional difficulties with the restrictiveness of the approach, resultant anxiety over blood glucose fluctuations with deviations from the eating plan, burden of management in different settings and the need for certain adjustments in lifestyle. In general, concerns over dietary restrictiveness may be one factor that limits widespread adoption of a VLC approach among patients with T1D, especially in children. One study found that caregivers of children with T1D expressed the desire to avoid limiting their child’s dietary intake or making them ‘feel different’ as barriers to healthful eating,^34^ and this may be even more pronounced with a VLC approach. This also raises the question about the sustainability of the VLC approach, which was a concern identified by a few DMPs and alluded to by some adult patients and caregivers of children with T1D (i.e. when describing deviations from the approach). Prior studies, particularly in T2D, indicate challenges with adherence to a low-carbohydrate eating plan.^35,36^ Further studies assessing the long-term sustainability of the VLC approach in patients with T1D, especially children, are needed. Furthermore, the appropriateness of a VLC approach for patients with T1D should be weighed carefully against these other considerations, and appropriate resources should be made available to those who decide to adopt this strategy to improve adherence rates.

Another key theme across responses from all the participant groups was the inconsistent support for a VLC approach within the medical community and its potential impact on patient- or family-provider rapport. From the patient and caregiver perspective, while some described supportive interactions, the predominant perception was limited provider understanding of and support for the VLC approach, oftentimes leading to misaligned goals of care and fractured relationships. Diabetes medical providers (DMPs), on the other hand, tended to lean more towards expressing support for the VLC approach, even amid concern for adverse sequelae, and described cooperative relationships with their patients and families. However, a number did express concern over suboptimal communication, and several voiced a desire for a more collaborative approach to care. This discrepant perception of the patient- or family–provider relationship by: (1) adult patients and caregivers of children with T1D, versus (2) DMPs, also evident on quantitative analysis of the data,^13^ may be partially explained by the latter group’s selection and social desirability biases. Diabetes medical providers (DMPs) who chose to complete the surveys and provide free-text responses (*n* = 61) represented only a fraction of all the adult patients and caregivers of children with T1D included in the study. Thus, those who decided to provide responses may have wanted to answer the questions in a way that would have been perceived more favourably, which is not uncommon in qualitative health research.^37^ The small DMP sample size included in this study may have represented biased provider perspectives and weakened conclusions regarding the observed discordance between: (1) patients and families, and (2) DMPs.

It is also important to note that a number of adult patients and caregivers of children with T1D admitted to limited sharing of information with the medical team, oftentimes because of prior negative experiences and, at times, fear of repercussions. As a result, not all DMPs were privy to their patients’ dietary strategies, and the survey questions for this cohort specifically did not mention the VLC approach to avoid disclosing this information. This may have impacted participant interpretation of the questions and affected which DMPs chose to respond and how they answered. To address the latter, in addition to considering all responses, we also stratified and analysed data from DMPs who had directly acknowledged or explicitly alluded to the VLC approach.

Regardless of the directionality of the perceived support, it is clear that there is a misalignment in the assessment of the status of the rapport between: (1) patients and families, and (2) DMPs, which deserves further attention.

Prior literature has suggested that effective medical provider–patient communications and trust in healthcare professionals have positive effects on patient outcomes.^38,39,40,41,42,43,44,45,46,47^ These observations have also been seen among patients with diabetes, with better patient perceptions of their medical team and satisfaction with care being associated with a higher likelihood of HbA1c testing completion within the past year^48^ and having a lower HbA1c.^49^ Conversely, the presence of mistrust in provider–patient relationships has been associated with negative patient outcomes, including those related to health symptoms and adherence to care.^46,47,50,51^ These concepts are important to consider within the context of this study, as the theme of subtherapeutic patient- or family–provider relationships predominated throughout participant responses, as discussed above. This included selective sharing of information by patients and families, reliance on providers solely for prescriptions and screening tests and interruption or transfer of care. These behaviours could have negative consequences for patient care, potentially depriving patients and families of the necessary support and guidance, ultimately leading to negative patient outcomes. Thus, efforts to optimise relationships between: (1) adult patients and caregivers of children with T1D following the VCL approach, and (2) the medical team, are critically important to ensure overall patient health and well-being. Recent experiences with healthcare models informed by carbohydrate restriction in other disease states, specifically prediabetes and T2D and other conditions involved in metabolic syndrome, have provided invaluable insights into care implementation strategies of this dietary approach and the perceptions of medical providers involved in these patient-centred initiatives.^52,53^ These can serve as important stepping stones for the design of similar interventions in the T1D population, informed by efforts to ensure both DMP engagement and support for the patients and families.

To further increase support for the VLC approach, patients and caregivers of children with T1D proposed several methods for promoting its awareness and understanding and facilitating its uptake. A large emphasis was placed on revamping the education for: (1) the medical community, and subsequently for (2) patients and families, the latter group ideally at the time of T1D diagnosis. As one way to foster this education, a call for more research was emphasised. Additional recommended venues for diffusion of information included mass and social media, community events and testimonials. Provision of resources, dissemination of Dr. Bernstein’s teaching and increased inclusivity and flexibility of the TypeOneGrit community were also noted as methods to facilitate adoption of a VLC approach. Finally, garnering support from the medical community, including getting endorsement from professional organisations, was highlighted as an important means by which to enable more widespread acceptance of a VLC approach as an adjunctive dietary strategy for T1D management. A minority of adult patients and caregivers of children with T1D, however, expressed scepticism about the ability of the VLC approach to reach a wider audience, emphasising the need for intrinsic motivation and the presence of external barriers. As discussed previously, while more studies describing the benefits of the VLC approach in T1D have been published in recent years since survey completion, concern over its safety and sustainability remains, thus limiting its acknowledgement as a viable dietary strategy by the medical community. This highlights the need for the dissemination of available patient, family and DMP experiences to reignite discussions regarding the role of the VLC approach in T1D management.

This study has several limitations, most importantly related to relevance and generalisability. For one, the survey study was conducted in 2016, and diabetes management has changed significantly since then, including increased uptake of diabetes technologies,^54^ which has led to improvements in glycaemic outcomes and quality of life.^54,55,56,57,58,59,60,61,62^ Perceptions and experiences regarding dietary approaches in this technology-dominated space may have changed.^63,64^ Additionally, there may have been a shift in the overall acceptance of the VLC approach as an adjunctive strategy for T1D management by the medical community over the past several years. Nevertheless, stakeholder perceptions on this topic have not previously been shared within the context of T1D and provide novel and valuable insights. To this day, while recognising the importance of carbohydrate monitoring and quality in T1D management, guidelines by major professional diabetes organisations still generally recommend ~40% – 50% of calories to come from carbohydrates.^2,3^ Thus, while initiatives to solicit updated and evolving sentiments regarding the VLC approach by patients, families and DMPs should be undertaken, the current findings still provide relevant insights into their experiences with this dietary strategy that should inform future studies and efforts to influence clinical guidelines.

Another limitation is that the original study had 316 international participants,^13^ 272 of whom were included in this qualitative analysis. This number represents only a fraction of all patients with T1D worldwide and likely a small percentage of those who have previously tried or currently follow a VLC approach. In addition, this study targeted a self-selected group of adult patients and caregivers of children with T1D who are members of the Facebook group TypeOneGrit, which is specifically designed for individuals who subscribe to Dr. Bernstein’s VLC approach for diabetes management, thus introducing selection bias. These individuals have chosen to subscribe to a VLC approach and, for inclusion purposes of this study, have followed this dietary strategy for at least 3 months. Thus, the perceptions and experiences of these participants with regard to the VLC approach itself are expected to overall be more positive, especially as compared to those who may have tried this eating plan for a shorter period, and may underrepresent neutral or adverse outcomes, thereby limiting generalisability. Moreover, this study did not assess the perceptions of children with T1D following the VLC approach. Thus, at this time, it is unclear whether the perceived benefits and acceptability of the VLC approach shared by the caregivers of children with T1D following this strategy also reflect the experiences of the youth. Future efforts should be undertaken to capture direct input from the children themselves to more accurately inform clinical and ethical considerations, especially as the latter pertain to concern for potential side effects more common to this population (e.g. impaired growth, restricted eating).

It is important to note that even the adult patients and caregivers of children with T1D that took part in this study did describe challenges with the VLC approach, in addition to its positive attributes, suggesting diversity of views. Furthermore, one major advantage of qualitative research, particularly when informed by phenomenological principles, is that it describes the lived experience of the participants.^20,21^ This provides invaluable insights about the phenomenon of interest (i.e. VLC approach in this case) that cannot simply be obtained from other research designs and quantitative data. However, especially when safety concerns are considered, it is important to complement these insights with objective data. It is important to note that in the parent study, while the primary data collection was via self-report, about half of the surveyed participants also provided objective information (e.g. regarding acute complications, HbA1c and other glycaemic parameters and lipids) by allowing the research team to survey their DMPs or by submitting medical records. Sensitivity analyses confirmed high concordance between self-reported and objective information within these participants, as well as between participants who did and did not provide this additional access.^13^ Regardless, more rigorous, long-term studies, such as RCTs, are needed to confirm these findings and to provide support for the safety of the VLC approach in T1D with regard to other parameters, such as cognition and growth in children.

## Conclusion

In conclusion, this qualitative study provided an opportunity to gain insights into the perceptions and experiences of patients, caregivers of children with T1D and DMPs with regard to a VLC approach for diabetes management. While describing many positive attributes of this dietary strategy for improving glycaemic outcomes, diabetes management and overall health, challenges were also conveyed, especially limited perceived support from the medical community. In addition to robust studies assessing the efficacy and safety of a VLC approach in both children and adults with T1D, efforts to improve relationships between: (1) patients and families, and (2) DMPs are paramount, especially as this dietary strategy gains popularity.

## Figures and Tables

**TABLE 1:** Numbers of coded responses per question by participant type.

Question	Adult patients with T1D (Q1-Q4) or their DMPs (Q5-Q6)	Caregivers of children with T1D (Q1-Q4) or children’s DMPs (Q5-Q6)	Total responses
Q1	32	36	68
Q2	114	91	205
Q3	123	94	217
Q4	116	85	201
Q5	31	24	55
Q6	11	18	29

DMP, diabetes medical provider; T1D, type 1 diabetes.

## Data Availability

The data that support the findings of this study are available from the corresponding author, Svetlana Azova, upon reasonable request.

## BOX 1: Open-ended questions included in the survey exploring patient, family and diabetes medical provider perceptions and experiences regarding a VLC approach in the management of type 1 diabetes.

### Questions for adult patients and caregivers of children with T1D

Q1. Why do you not discuss your diet with your doctor? (*If responded ‘No’ to the question, ‘Do you openly discuss your diet and other aspects of the GRIT approach with your professional diabetes care provider?’*)
Q2. What else would you like to tell us about your own experiences following the low-carbohydrate diet?
Q3. Please tell us about your experiences with your diabetes health care providers.
Q4. How do you think the GRIT approach might be expanded to a larger audience?

### Questions for diabetes medical providers

Q5. Please comment on how you feel about your patient’s diabetes management approach.
Q6. What else would you like to tell us?

Note: GRIT refers to the approach for managing type 1 diabetes via a very low-carbohydrate diet.

T1D, type 1 diabetes; VLC, very low carbohydrate.
